# Motherhood: Female Perspectives and Experiences of Being a Parent with ASC

**DOI:** 10.1007/s10803-021-05122-5

**Published:** 2021-06-09

**Authors:** Rebecca Winnard, Mark Roy, Hannah Butler-Coyne

**Affiliations:** 1 School of Psychology, University of Central Lancashire, Darwin Building, Preston, PR1 2HE UK; 2 Tier 4 CAMHS Inpatients Services, Lancashire Care NHS Foundation Trust, Piccadilly, Lancaster, LA1 4PW UK

**Keywords:** Autistic spectrum conditions, Parenting, Interpretative phenomenological analysis

## Abstract

Little is known about the emotional pressures and practical management of daily challenges and, intra and interpersonal demands of raising a child as a parent with a diagnosis of Autistic Spectrum Conditions. The present study utilised a qualitative approach to understand perceptions of females diagnosed on the autistic spectrum of ‘being a parent’. Eight semi-structured interviews were analysed using Interpretative Phenomenological Analysis. Benefits and challenges of being a parent were highlighted alongside population-specific skill and characteristics associated with strength and resilience, love, nurture, routine and sensory considerations. Findings identify the need for population-specific specialist parenting support, provide direction for professionals in clinical settings and expand the paucity of research in this area.

## Introduction

Parenthood is arguably one of the most significant life events an individual and family can experience, often leading to a multitude of new responsibilities alongside a range of physical, psychological and social changes (Davies & Harman, 2017). Parental responsibilities inherently result in additional pressures with individuals negotiating the management of (new) household and childcare tasks, balancing (pre-existing) work positions, establishing the parent–child relationship whilst also adjusting to altered family (and life cycle) roles (Khajehei, 2016; McGolrick et al., 2011; Mosek-Eilon et al., 2013). Parenting literature acknowledges the realistic strains and pressures associated with developing the parent–child relationship with the parental role being specifically impacted by sleep deprivation (Medina et al., 2009), finances, marital relationship quality (Doss et al., 2009) and psychological wellbeing with an increased vulnerability to mental health difficulties (i.e., post-natal depression and anxiety, Grant et al., 2008; Ierardi et al., 2019; Korja et al., 2015).

Various pieces of advice are often offered via differing forums by professionals, friends, (social) media and, more generally, the societal and cultural expectations about what constitutes as ‘good and bad parenting’ (Olsen & Clarke, 2003; Riggs et al., 2016; Weigel, 2008). Generally, ‘good parenting’ is described as the individual being a positive role model to their children and other parents, offering warm and loving support, making life choices that do not impede the child’s emotional, social and physical development, providing stability, nurture, and close supervision (Asmussen, 2011; Couvrette et al., 2016; Sandstrom & Huerta, 2013). Alternatively, inconsistent and unpredictable parenting approaches are often stated as elements of ‘bad parenting’ (Nanninga et al., 2015) with academic evidence highlighting this parenting style as having significant consequences and impact upon cognitive development and functioning (Evans et al., 2011; Shonkoff & Garner, 2011), understanding and management of emotions (George et al., 2017; Hudson & Jacques, 2014; Sandstrom & Huerta, 2013) and ultimately mental health and behaviour (Asmussen, 2011). Equally, expectations of parenting practices can be further complicated and influenced by cultural and sub-cultural values and practices (Connolly & Ward, 2008) as well as societal and (inter-generational) attitudes as to what constitutes as ‘good and bad parenting’ whilst also an ‘ideal’ family (e.g., same sex parents, Layner et al., 2014; parents with a disability, LaLiberte et al., 2017; parents with mental health difficulties, Fatemeh et al., 2011).

The challenges of parenting are complex with different parent populations evidently encountering and negotiating similar yet also, specific multifaceted elements. Despite ample research offering insight into general parenting experiences (Fillo et al., 2015; Larsson et al., 2015; Mickelson & Biehle, 2017) and the transition into parenthood (e.g., same sex parents, Layner et al., 2014; parents with physical health difficulties, Jabbar, 2014; deaf parents, Moroe & Andrade, 2018; parents with severe mental illness, Tabak et al., 2016; parents with a learning disability, Pethica & Bigham, 2018) there is a paucity of research exploring the experiences of parenthood for those individuals with neurodevelopmental difficulties, namely, Autistic Spectrum Conditions [(ASC); Hendrickx, 2015; Olsen & Clarke, 2003]. A high proportion of women receive a late diagnosis of ASC with this often at a time when they have already started a family will be, therefore, negotiating parenthood with either little support or by navigating the advice from generic parenting programmes (Loomes et al., 2017). Research in this area is imperative in understanding the difficulties this population of mothers encounter in order to make reasonable adjustments to support women with a diagnosis of ASC during pregnancy and subsequent motherhood (Grant, 2015). Previous research conducted with specific populations identified key themes highlighting possible benefits and challenges of parenting. The importance of establishing a positive relationship with the child was recognised, alongside providing a nurturing environment aiding development (parents with a learning disability, Sheldon et al., 2019, Macintyre & Stewart, 2012; parents with severe mental health difficulties, Bartsch et al., 2016, Nilova et al., 2017). Accessing the right support regarding the transition to parenthood and the anxiety of socialising with other parents and teachers was highlighted as a challenge (parents with a learning disability, Sheldon et al., 2019, Macintyre & Stewart, 2012; parents with mental health difficulties, Bartsch et al., 2016; Nilova et al., 2017; deaf parents, Moroe & Andrade, 2018; same sex parents, Layner et al., 2014).

With a ratio of more than one in a hundred people, it is estimated that around 700,000 people in the United Kingdom have a diagnosis of ASC (National Autistic Society [NAS] 2019; Royal College of psychiatrists [RCPSYCH] 2019). Generally, individuals with a diagnosis of ASC experience challenges in three main areas (e.g., the triad of impairment, Wing & Gould, 1979); social communication (verbal and non-verbal), social interaction and social imagination (Attwood, 2008; Gernsbacher & Pripas-Kapit, 2012; Moreno et al., 2012; Wing & Gould, 1979) alongside sensory sensitivities (Diagnostic and Statistical Manual of Mental Disorders, fifth edition, [DSM-V] 2013). In consideration of the difference in world view and specific daily challenges those individuals with ASC are negotiating, it is not surprising that the limited research in this area identifies various perceived and encountered challenges. Interpretation of complex personal emotional experiences and understanding those of others (Golan et al., 2015) whilst also negotiating and managing large quantities of information (particularly from professionals) alongside deciphering the intricacies of social interaction were all deemed as extremely challenging, stressful and prohibiting to being able to fully explain and address their child’s needs (Simone, 2012; Attwood & Grandin, 2006; Attwood, 2008; Hendrickx, 2015; Riley-Hall, 2012; Schopler & Mesibov, 2013). Equally, the basic sensory and practical demands of raising children are cited as extremely difficult to manage due to a particular susceptibility to sensory overload (Grant, 2015; Hendrickx, 2015; Mendes, 2015; Moreno et al., 2012, Willey, 1999) and adjustment challenges associated with change to pre-existing routines (Hendrickx, 2015). For those parents with a diagnosis of ASC, the apparent additional challenges with adapting to parenthood can impact on their psychological wellbeing and mental health (Aston, 2002). A lack of tailored support and resources for individuals with ASC, including parenting resources, overlooks the specific needs of this parent population and highlights a significant gap in knowledge as a result of a neglected research area (Dissanyake et al., 2020).

Anecdotal evidence as to the experiences of parenting for mothers with ASC appears to currently outweigh the research literature in this area (see Grant, 2015; Hendrickx, 2015; Willey, 1999). The limited empirical research exploring this area has evidently resulted in insufficient informed guidance, policy and essential documentation for both health and social care settings and practitioners as to how best support and tailor interventions for this sub-population of parents. With this in mind, and the tangible need for further in-depth investigation, the main purpose of this research is to develop understanding of experiential and prospective concerns, challenges and benefits for mothers with a diagnosis of ASC. Research questions to scaffold the overarching aim are;

- How do individuals with ASC experience, or perceive they will experience, being a parent?
- Do particular characteristics associated with ASC compliment or complicate parenting experiences?
- How can parenting support be tailored to support this population?

## Method

### Ethical Considerations

The study was reviewed and approved by the Ethics Committee, University of XXX. As per British Psychological Society Code of Ethics and Conduct (2018), written informed consent was obtained from all study participants.

### Methodological and Data Analysis Approach

A qualitative research methodology employing a semi-structured interview data collection format and utilising Interpretative phenomenological analysis (IPA) was deemed the most appropriate approach to gather subjective experiences, perspectives and meaning associated with parenting for this population (van Schalkwyk & Dewinter, 2020; Curry et al., 2009).

IPA draws upon the philosophical foundations of phenomenology, hermeneutics and idiography to identify the components of individual’s lived experiences, developing understanding through both intra-subjective and inter-subjective meaning (Alase, 2017; Freeman, 2008; Jachyra et al., 2018; Pietkiewicz & Smith, 2014). This study used a dual hermeneutic method, analysing individual’s perspectives regarding the social world, as this is deemed a valuable technique when conducting studies that have been under-researched and lack an empirical, conceptual or substantive basis (Jachyra et al., 2018).

### Sampling and Recruitment

A dual recruitment approach was employed to optimise opportunity to gather a range of perspectives regarding opinions of, and parenting experiences for, the ASC population. Female participants with a diagnosis of ASC were recruited using purposive snowball sampling across two recruitment approaches; advertisement in an Autism Support Group in the North of England and, through an Autism Parenting Group on social media.

Eligibility criteria to take part in the study were; 18 years old or over, identified as of female gender, received a formal diagnosis of Autistic Spectrum Conditions (as per DSM-V criteria, APA, 2013) and, understanding of, and verbal fluency, in English.

To enable a holistic view of the topic, prevent bias of participant opinion and influence of age/point in the life cycle, during the early stages of exploring this topic area, both parents and non-parents with a diagnosis of ASC were recruited to take part in the study.

### Participants

A total of eight females aged between 28 and 63 years old (mean = 41.5; SD = 12.3) took part in the study. Participants reported receiving a diagnosis of ASC between 9 and 50 years old (mean = 28.9; SD = 14.9), with only one participant receiving a diagnosis as a child. Participants lived across England (n = 7) and Scotland (n = 1).

Four participants were parents (mean = 51.3; SD = 7.4), with children aged between 8 and 40 years old (mean = 23; SD = 8.9). Three participants had children who were diagnosed with autism with one participant also having two children without a diagnosis of autism. Initially, all participants had partners that could support with childcare. At the time of the research interview, two participants had separated from their children’s father.

The remaining four participants were not parents (mean = 31.5; SD = 3.5) who had no parenting experience. Only one participant expressed a desire to be a parent.

### Interview Protocol and Procedure

A semi-structured interview guide was developed through review of existing literature (e.g., DePape & Lindsay, 2015; Werner-Bierwisch et al., 2018) and discussion with the research team, a Health Psychologist and Clinical Psychologist who had experience in the area yet were not blind to the research hypothesis.

The interview comprised four broad sections with nine questions in total (two questions for each of the first three sections and three questions for the fourth), all designed to be open-ended and flexible, as per IPA approach and ethos. Section and example questions included:

1. The Meaning of Parenting: ‘*What does being a parent mean to you*?’
2. Benefits: ‘*Tell me about what you think are the ‘good things’ about being a parent’*
3. Challenges: ‘*What do you think are the most challenging aspects of being a parent?*’
4. ASC Parenting Idiosyncrasies: *‘Do you think having ASC impacts upon how individuals’ parent?’*

Interviews took place at the venue used for the autism support group or via telephone due to participant preference or to accommodate logistical challenges. To ensure a standardised approach, interviews were conducted by the Lead Researcher who had experience in qualitative methodology. All interviews were audio-recorded, transcribed verbatim and anonymised, and lasted between 15 and 60 min in duration. Data collection took place over a 6 month period.

### Data Analysis

Data analysis was conducted over four stages as outlined by the IPA procedure (Pietkiewicz & Smith, 2014).

To ensure full engagement with the data, interviews were recurrently read, with key elements of personal perceptions of living with a specific condition or being in a particular situation initially drawn out (Biggerstaff & Thompson, 2008; Stage 1). Content and language were analysed with the development of possible meanings and interpretations and noted annotations. Conceptual similarities and reoccurring themes where identified across the interviews (Pietkiewicz & Smith, 2014; Stage 2). Observations from each interview were compared and patterns were identified, displaying interrelationships between the interviewees (Jachyra et al., 2018), and in doing so developing key themes. Super-ordinate and subordinate themes, true to the key content were drawn (Stage 3), highlighting similarities between interviewees experiences in relation to the questions asked. Lastly, themes were reviewed to ensure close links with the raw data and are reported in this paper (Stage 4).

### Reflexive Positioning

The IPA method relies on the researcher’s interpretations and analysis of the participant’s experiences to be situated from an objective standpoint. Reflexivity requires recognition and self-reflection of the impact the researchers social background, preconceptions and behaviour have upon the research process (McCabe & Holmes, 2009). The Lead Researcher reflected on their pre-existing position of working as an Assistant Psychologist within an adult autism assessment service. Equally, the research team, a Health Psychologist and a Clinical Psychologist, at various points throughout the research process, reviewed their position alongside the data, to ensure transparency and openness as to conflicts and positioning. The research team engaged in continual critical and conscientious evaluation regarding positions in relation to the individuals participating, to increase awareness of influential positions (Bishop & Shepherd, 2011). Whilst analysing the data the Lead Researcher used bracketing which sets aside thoughts about the phenomenon (King, 2004). A reflective dialogue was documented as part of all research supervisory meetings to further enable transparency of researcher influence and offer reflective opportunity to enable transferable and verifiable research findings (Mauthner & Doucet, 2003). Validation checks were conducted on the analysis of the transcripts and sub-ordinate themes by the research team, to corroborate representativeness and reflexivity (Engward & Davis, 2015). Quotes were also used to amplify points, safeguard authenticity whilst also connect the reader to the participant and population voice (Larkin et al., 2006). Aside from ensuring trustworthiness, member-checking, triangulation and, continual evaluation as part of assuring quality in the research approach and data (Alase, 2016), a quality and verification tool was adopted to further safeguard standards and credibility (Alase, 2017).

## Results

Cross sub-population (e.g., parents [*P*] and non-parents [*NP*]) consistent theme commonalities were found within the data. Six superordinate themes were identified; ‘*Parenthood: Fun & Games*’, ‘*Support: Giving & Receiving*’, ‘*Routine & Structure*’, ‘*Sensory Sensitivities*’, ‘*Interaction*’ and ‘*Unique Insight*’. Participants are assigned a pseudonym to maximise confidentiality (Baxter et al., 2010) with quotations included to illustrate and describe themes (see in-text descriptions).

### Superordinate Theme 1: Parenthood: Fun and Games

A positive parent–child relationship was reported as important for providing, sharing and receiving ‘love’:

*“Well good things about being a parent is the absolute love you get from your children and the love you give to your children and what you can share with them”* Chiara [*P*]

The concept of ‘love’ included viewpoints referring to the emotional experience of “*instant*” feelings of love and connectedness towards their child. Participants commented on the demonstration and display of love being more about the process of meeting practical needs and ‘task-focused’ nature:

*“Making sure they know right from wrong and are safe, erm they don’t get into trouble. Support them, erm, with things they want to do. It might involve things they want to do like going swimming or anything they want to do. (Pause) Looking after them by making sure they have what they need and go to school*.” Hannah [*NP*]

More generally, ‘spending time’ and ‘being’ with their child(ren) was deemed as enjoyable and fun aspects of parenting, with reports of being able to reconnect to their own child ‘self’ through this process:

“*Being a big kid again. Doing things like dressing up for Halloween and things like that and taking them on family holidays”* Catherine [*NP*]

### Superordinate Theme 2: Support: Giving and Receiving

Experience, generated knowledge and various forms of support for the parent and child were highlighted as important platforms to guide parents understanding of parenting. The achievement of practical and task-orientated responsibilities when supporting a child to learn, develop and understand the world were referenced as essential observable factors associated with the rewards of parenting:

*“I get a lot of joy from the way their brains work, we unpick the day and unpick situations and we work it out together”* Chiara [*P*]

*“When you are growing your own kids and seeing all these things it feeds into you as well and makes you want to be, makes you want to do better”* Amy [*P*]

Difficulty accessing specialist support for parenting and their children was identified as being a particular challenge for parents with a diagnosis of ASC. Significant struggles and frustrations were reported when attempting to access services to support their children’s needs, particularly for those parents that recognised their children to have traits of ASC.

*“I have to fight my goodness I have to fight for them, especially my son and the local authority and getting a diagnosis and things like that”* Chiara [*P*]

### Superordinate Theme 3: Routine and Structure

The majority of participants, regardless of being a ‘parent’ or ‘non-parent’, expressed that parents with a diagnosis of ASC have remarkable skills in ensuring routine and structure, factors deemed extremely beneficial for both parent and child:

“*For me I suppose one of my biggest traits is that I need structure and I need routine and obviously that helps kids, well the evidence is kid’s kind of prefer structure and routine”* Jade [*P*]

Equally, parents with ASC were reported to be able to “*positively and helpfully*” impress routine and structure within a child’s daily routine more efficiently and effectively than parents without a diagnosis of ASC, due to their own innate strive for structure. However, being flexible to, and managing the multiple and often changing, unpredictable parenting tasks alongside other demands was referred to as a specific challenge for this population of parents.

“*I just couldn’t multi-task and have a work head and a parent head because I have to be all consuming in what I am doing*” Chiara [*P*]

For NP participants, the majority described feeling daunted and concerned about the potential changes a child would bring to their routine and schedules.

*“I like my routine. I like to know what is happening and when. I don’t like things suddenly changing”* Sarah [*NP*]

### Superordinate Theme 4: Sensory Sensitivities

A common view was the perceived personal sensory sensitivities causing, or having the potential to cause, difficulties in being a parent:

*“I am sensitive to noise and visual stuff and there are kids screaming, there are balloons popping, there is music and banging about”* Kirsty [*NP*]

Parent participants tended to recall tactile and olfactory challenges particularly when preparing food and engaging in play:

*“I have lots of sensory issues around food and I am having to have almost my own mini meltdowns of hell preparing all this food”* Chiara [*P*]

*“[I am] not doing things that are arty and crafty and not cooking stuff with them because I can’t stand mess and dirt”* Jade [*P*]

Consequences of managing sensory overload were described as having to cope with significant amounts of parenting-specific ‘stress’ and anxiety on a daily basis. However, regardless of these concerns there were no specific statements associated with not wanting to be (future) parents.

### Superordinate Theme 5: Interaction

A variety of new challenging social situations were acknowledged as a result of parenthood, with particular emphasis on the difficulties associated with communicating with teachers, being overwhelmed with verbal information, ensuring that they are understood by others and more generally, engaging in education protocol (e.g., parent’s evenings etc.):

*“…it might be difficult for the parent to understand what the teacher is saying, especially if it is all verbal. It might be difficult to ask the right questions about their child”* Kirsty [*NP*]

Social interactions with other parents and children were referred to as mandatory yet described as feeling forced, unpredictable and a particularly challenging aspect of being a parent with ASC:

*“The school thing was a nightmare because that made me make contact with other parents, which I had to avoid like the plague because I didn’t want to get, because I never, because you are totally unscripted”* Amy [*P*]

Interaction with others, as a consequence of extracurricular activities and school peer group, was identified as a daunting experience, often met by parents wanting to avoid the situation. A parent’s resistance to social interaction was acknowledged as being impactful on children’s social functioning:

*“…they didn’t really play with their friends that much outside of the school. But then I suppose, whether they could pick up on the fact I didn’t want to do it I don’t know”* Jade [*P*]

### Superordinate Theme 6: Unique Insight

Participants described being a parent with ASC as an advantage of parenting a child who had the same diagnosis. The unique insight and ability to ‘truly’ relate as a result of their personal experiences and anecdotal evidence was deemed as key in providing a level of parenting only achievable by those with a diagnosis of ASC.

Participants commented on being an ‘expert-by-experience’ and using this specialist insight and knowledge as a foundation to support their own children with ASC, by employing more individualised ASC knowledgeable strategies and interaction techniques:

*“…it might be that they have better ideas on bringing them up and might interact with them better ironically because they are not doing what everyone else is doing and trying to follow the crowd*” Catherine [*NP*]

Equally, being able to understand and compartmentalise the difficulties pertinent to ASC separate to a child’s own identity was referred to as an important factor in empowering and promoting individuality.

*“Just get to know your kid and get to know them as an individual and deal with their stuff not everything that you read in a book”* Amy [*P*]

## Discussion

To the authors’ knowledge, this research is one of few studies exploring the perspectives and experiences of parenting and being a parent among females with a diagnosis of ASC. Strong theme commonalities were found across the sub-populations (e.g., parents and non-parents) with six superordinate themes identified and consensus as to the possible benefits and challenges of being a parent with ASC.

The need for, and adherence to, structure and routine, as experienced by parents diagnosed with ASC, was suggested as being an advantage to parenting children and a technique this population of parents were particularly skilled (Shore & Rastelli, 2011; Hendrickx, 2015). Equally, routine and structure are cited in various parenting literature as being of significant benefit for children to establish familiarity, consistency and in developing time management skills (Markham, 2014; Miller, 2018). However, it was acknowledged that the routines and at times unpredictability aligned with parenting may disrupt pre-parenthood structure and in doing so cause stress and anxiety (Hendrickx, 2015), congruent to the general population’s anxieties regarding parenthood (Mihelic et al., 2018).

Social interaction was highlighted as a challenge for this population of parents with identified difficulties, frustration and anxieties in communicating with teachers, other parents and their children’s friends whilst also in negotiating education and school engagements and impromptu meetings (i.e., school gate conversations). Social encounters were deemed as being extremely effortful and a possible reflection of well documented identified communicative difficulties for this population (i.e., challenges with initiating conversation, understanding abstract language and body language, APA, 2013; Levy & Perry, 2011; Müller et al., 2008). Resultantly, the potential investment of energy into attempts to ‘mask’ experiences of stress, and ‘hide’ behaviours and/or develop coping strategies such as social camouflaging (Lai et al., 2017; Schneid & Raz, 2020) and impression management (i.e., utilising strategies to influence how others perceive the individual; Hull et al., 2017) may have further contributed to stress and difficulties. Additionally, the unpredictable nature of an ‘interaction’ was referred to as concerning with acknowledgement that situations may be actively avoided and as such impact on their children’s social contact at times (Hendrickx, 2015; Moreno et al., 2012; Simone, 2012). Factors associated with social inclusion and communication challenges for individuals with ASC when in predominantly ‘typically developing’ or ‘real world’ environments (such as mainstream schools) is under-researched. Limited existing literature identifies social interaction and outcomes as dependent on the ‘fit’ between the individual and the social environment, with social affiliation increasing when interacting with other individuals with ASC, suggesting autistic sociability is a relational difficulty rather than individual challenge (Morrison et al., 2020).

Sensory overload and difficulties in navigating the various sensory demands children bring to everyday life was identified as providing specific practical and emotional challenges for parents with a diagnosis of ASC. Consequently, individuals with sensory sensitivities who are parents are managing *extra* sensory demands which can potentially cause stress, anxiety and physical pain and hence, impact on psychological wellbeing and mental health (Heffernan, 2016; Jurkevythz et al., 2020). Parents with a diagnosis of ASC may need more down time from situations which are particularly ‘sensory rich’ such as a child’s tantrum, sibling fights, children’s television and noise in order to prevent sensory overload (Simone, 2012).

Access to support in both educational and healthcare settings was considered a “*fight*”. Challenges encountered with social interaction and communication for this parenting population is referenced within this study and previous research, and as such highlights how these difficulties may contribute to providing barriers in being able to access the right and most appropriate (specialist) support (Attwood & Grandin, 2006).

As supported by pre-existing research and anecdotal evidence (Crane et al., 2021; Hendrickx, 2015; Simone, 2012), a key benefit to being a parent of a child with the same ASC diagnosis was a resultant unique insight, bond and level of understanding of the experiences. As such, individuals highlighted an affiliation and ability to being able to offer truly informed individualised and experiential driven understanding, strategies and techniques for challenges. Unique insight and knowledge held by the ASC parenting population is arguably an advantage when parenting children with ASC with literature highlighting a ‘neurotypical’ parents adopted advocacy role associated with this scenario being challenged due to the energy and efforts required in ‘seeking self-education’ (Boshoff et al., 2016, 2018). Equally, the acceptance of differences and promotion of individuality has been found to be stronger within the ASC parenting population when compared to the neurotypical population (Jurkevythz et al., 2020).

### Study Limitations

Participants consisted of both mothers and non-mothers providing a general view of the perceptions of parenthood with experience not being a limitation. As the research was conducted solely with women, the findings are not generalisable to all parents with a diagnosis of ASC and therefore, further research is needed to explore male perceptions and experiences of parenthood. Alongside this, the amount of support from partners and family is unknown, which could have impacted upon perceptions of the roles, responsibilities and pressures of parenting. More exploration of the impact/effect of this factor would have added further context to the data.

More generally, although the research team comprised professionals with experience in both parenting and working with individuals with an ASC diagnosis, there was a lack of valuable input and specialist expertise of ‘experts-by-experience’. Future investigations should ensure that ‘experts-by-experience’ are involved throughout all research phases of research (Di Lorito et al., 2018; Paul & Holt, 2017).

### Practice implications

Research considering parenthood for individuals on the autistic spectrum is limited, highlighting a gap in knowledge for this population of parents. As such, professionals, in both educational and health care settings are at risk of providing techniques, strategies and even manualised parenting approaches (e.g., Webster Stratton Incredible Years, Webster-Stratton, 2005; Triple P, Sanders et al., 2014; Mellow Parenting, Macbeth et al., 2015) without considering the specialist needs of this population of parents. Clinical practitioners and educational professionals need to reflect on and consider crucial reasonable adjustments for this parenting population (such as practicalities e.g., managing personal sensory sensitivities, length and time of appointments, visual supports, written information discussed within the session, social interaction anxieties/challenges) in order to enable optimum engagement and interaction. Equally, professionals should empower parents through focusing on pre-existing understanding and skills, encouraging attendance at parental education groups and peer support groups, as a means of promoting awareness and knowledge whilst also attempting to reduce stress (Dawson-Squibb et al., 2018; Stuttard et al., 2016). More generally, raising awareness and understanding within health and educational settings of the needs and challenges of all parent sub-populations (mental health, ASC, physical health etc.) would aid in supporting the care and provision of services to parents accessing them for their children.

### Future Research

As stereotypes and preconceptions of the abilities of individuals with mental and physical health difficulties change within society (STOMP [stopping over medication of people with a learning disability, autism or both] agenda, 2016a; Transforming Care agenda, 2015; The 5 year forward view for mental health, 2016b; Autism Strategy, 2015), more research is needed to highlight the resilience and strength of these individuals. Future research should consider both mothers and fathers experiences of being a parent with ASC in more detail, potentially aiding professional understanding, informing support strategies and challenging societal stereotypes. Additionally, further exploration into the specific support required for this parent population is needed, including the reported challenges and access to support networks, to enable the development of additional provision to achieve their potential.

## Conclusion

This study explored the perspectives and experiences of parenthood for females with a diagnosis of autism spectrum condition. The findings of this research highlighted a number of population specific benefits and challenges of being a parent with autism and in doing so contributes to and expands upon, the knowledge regarding this population; including the reasonable and practicable adjustments needed to improve the access to efficient and effective support, empowering skills to parent to the highest of their abilities, as provided to other parenting populations.
